# 

**Published:** 1897-07-24

**Authors:** 


					THE HOSPITAL. ABSOLUTELY PURE, ^therefore BEST. July 24th, 1897. (/
CADBURY'S o CADBURY'S
t
COCOA
" The standard of highest purity at present <?> i NO ALKALIES USED,
attainable."?Lancet.
COCOA
WlYICH HOSSIJ-AJL
NO. 5&. Vol. XXII. [f#?er!J Saturdat,
July 24th, 1897.
OUUBUlip
s6e p.280. J PRICE XWUri^

				

